# Lobular Mammary Carcinoma Presenting as an Obstructing Rectal Mass

**DOI:** 10.1155/2021/2416950

**Published:** 2021-11-20

**Authors:** Ebrahim Almahmeed, Eman Aljufairi, Noof Alshaibani

**Affiliations:** ^1^Department of General Surgery, King Hamad University Hospital, Bahrain; ^2^Department of Pathology, Blood Bank and Laboratory Medicine, King Hamad University Hospital, Bahrain

## Abstract

Breast cancer is the leading cause of cancer death in women, and while metastasis is common to areas like the bone, lungs, and brain, it is rare to metastasize to the gastrointestinal tract and especially to the rectum. Due to the rarity of this condition and its resemblance clinically and radiologically to primary gastrointestinal tract tumors, diagnosis and treatment are challenging. We present a case of metastatic lobular mammary carcinoma in a 52-year-old Bahraini woman who presented with an obstructing rectal mass.

## 1. Introduction

Breast cancer is the commonest cancer in females, affecting approximately 12% of women worldwide [1]. The 2 main histologic subtypes of primary breast cancer are ductal and lobular carcinoma, with the latter being the second most common which accounts for approximately 10% of all breast cancer cases [2].

Although there is improvement of the screening, diagnosis, and treatment of breast cancer, the development of distant metastases occurs and is the major cause of death in breast cancer patients [3]. Overall, the most common sites for distant metastasis are bone, lung, liver, and brain metastasis [4].

Gastrointestinal tract metastasis is rare with breast cancer and accounts for less than 1% of the cases, with rectal metastasis being an extremely rare entity [5].

We present a case of primary invasive lobular breast cancer presenting with rectal metastasis causing bowel obstruction in a 52-year-old Bahraini female.

## 2. Case Presentation

A 52-year-old female, who is a known case of diabetes mellitus on oral hypoglycemics, presented to the emergency department complaining of diffuse on and off abdominal pain, nausea, vomiting, and obstipation for 1 week.

The patient reported constipation for 1 month before it progressed to obstipation and unintentional 8 kg weigh loss over 3 months. She gave a history of total abdominal hysterectomy in 2015 due to uterine fibroids. The patient had no personal risk factors nor family history of malignancies.

Abdominal examination was unremarkable while the anorectal examination showed a circumferential rectal mass that is 8-9 cm from the anal verge. Initial laboratory investigations and abdominal X-ray were unremarkable.

**Figure 1:**
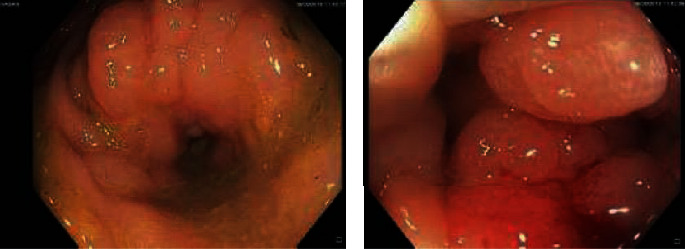
Colonoscopy findings of stenotic rectal mass.

**Figure 2:**
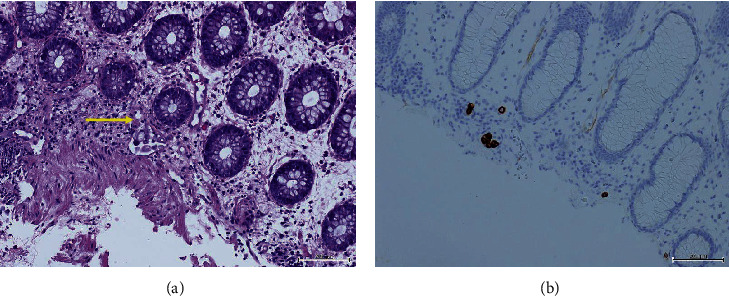
(a) Rectal biopsy showing signet ring cells (yellow arrow). (b) Rectal biopsy positive for cytokeratin 7 immunostain.

The patient underwent an urgent colonoscopy which showed a malignant-looking lesion with severe stenosis at the proximal rectum 10 cm from the anal verge (Figure 1). A biopsy was taken and revealed few signet ring cells with CK7 histochemical stains being positive while CK20 and CDX2 were negative (Figures 2(a) and 2(b)). A performed gastroscopy was unremarkable.

Further investigation with CT of the chest, abdomen, and pelvis; MRI of the pelvis; and whole body PET/CT was carried out.

**Figure 3:**
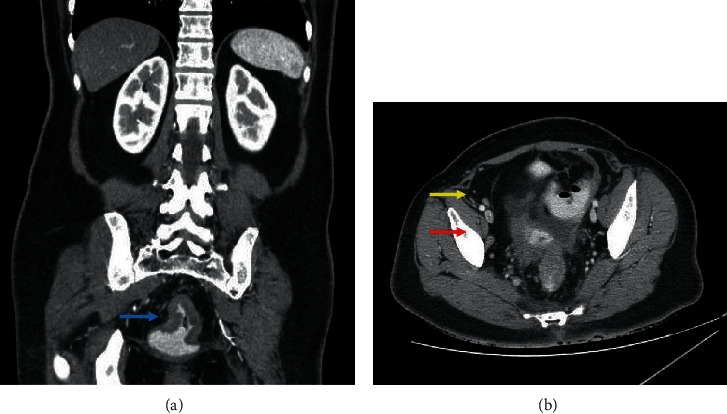
CT of the abdomen and pelvis in coronal view (a) showing a rectal mass and in axial view (b) showing small noncalcific right internal (red arrow) and right external (yellow arrow) iliac lymph nodes.

The chest CT showed multiple scattered focal lytic and sclerotic lower cervical, dorsal vertebral, rib cage, and sternal metastatic deposits, while the abdomen and pelvis CT with MRI of the pelvis revealed a circumferential heterogeneously enhancing rectal mass lesion (about 7 cm in length) located 7 cm above the anal verge, with a maximum wall thickening of about 1.75 cm, resulting in rectal luminal narrowing with multiple small noncalcific mesorectal, right internal, and right external iliac lymph nodes (Figure 3).

**Figure 4:**
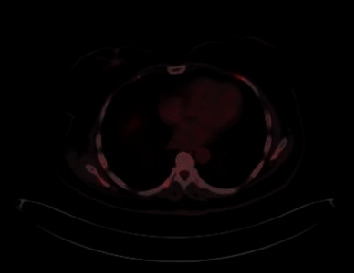
Whole body PET/CT showing right breast suspicious mass.

The whole body PET/CT showed the same findings of the CT and MRI previously done (rectal mass and multiple bony metastatic deposits) in addition to suspicious right breast upper inner quadrant lesion (Figure 4).

**Figure 5:**
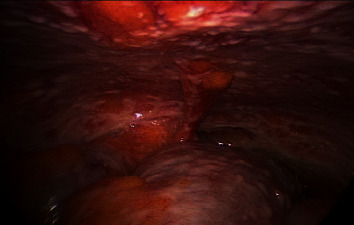
Diagnostic laparoscopy showing multiple peritoneal metastatic lesions.

**Figure 6:**
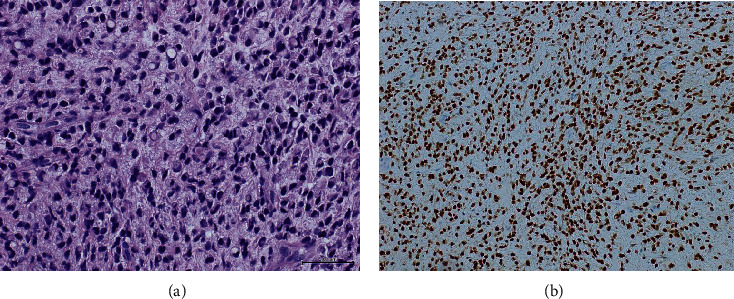
(a) Peritoneal deposit biopsy showing discohesive cells with plasmacytoid and signet ring morphology. (b) Peritoneal deposit biopsy showing sheets of malignant cells positive for estrogen receptors (ER).

In view of the almost complete bowel obstruction, the patient underwent laparoscopic diverting loop ileostomy to relieve the symptoms, which also showed disseminated peritoneal metastatic deposits (Figure 5) that were biopsied and turned out to be metastatic carcinoma, showing sheets of pleomorphic malignant signet ring-like cells on histopathology. CK7 and estrogen receptors (ER) were positive (Figures 6(a) and 6(b)) and CK20, GCDFP-15, E-cadherin, WT-1, and Her-2 Neu were negative on immunohistochemistry staining.

**Figure 7:**
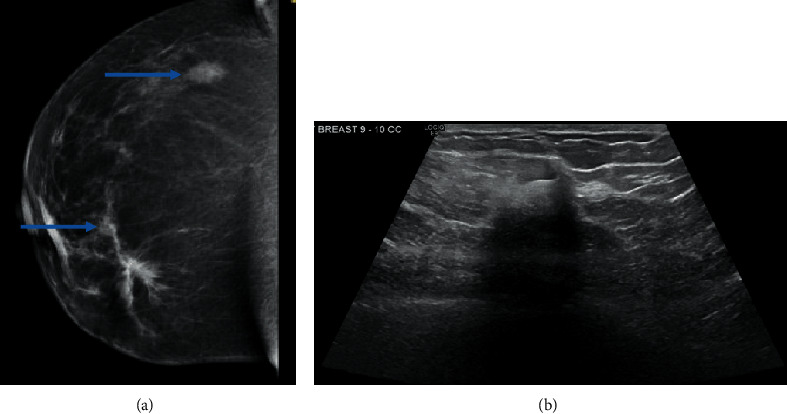
(a) Two lesions in the right breast on mammogram (blue arrows). (b) Right breast lesion.

The patient was further investigated for the right breast mass with ultrasonography and mammogram of the breast that showed 2 ill-defined, irregular lesions in the right breast (Figure 7).

**Figure 8:**
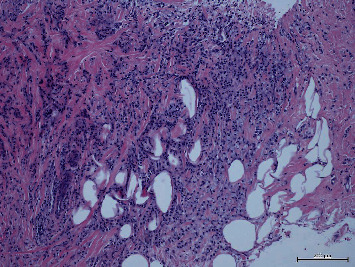
Right breast lesion biopsy showing invasive lobular carcinoma, arranged in sheets, cords, and small clusters.

An ultrasound-guided core biopsy of the right breast 10 o'clock lesion was taken (Figure 8), and the histopathology report came as invasive lobular carcinoma, grade III.

The immunohistochemistry and receptor status were positive for CK7—similar to the rectal mass results—ER receptor, and PR receptor, and it was negative for CK20, E-cadherin, and Her-2 Neu receptor, with a tumor proliferative index (by Ki-67) of 1%.

The final diagnosis was right breast invasive lobular carcinoma, grade III, T1NxM1, ER +ve, PR +ve, Her-2 Neu -ve, Ki-67 1%, luminal A subtype.

The patient's case was discussed in the national tumor board meeting and was planned for palliative chemotherapy and hormonal therapy.

## 3. Discussion

Metastatic tumors of the gastrointestinal tract (GI) are considered rare, and the commonest primary tumors metastasizing to the GI tract are melanoma and breast cancer [6]. In clinical practice, the percentage of primary breast malignancies metastasizing to the GI tract is less than 1% [7]. It was found that the small bowel and the stomach are the commonest site for metastasis, whereas rectal metastasis accounted for about 7% only [8].

Invasive lobular carcinoma is found to have a higher metastatic rate (4.5%) to the GI tract than invasive carcinoma of no special type (0.2%) [9], although the latter is the commonest breast cancer; the reason of more frequent spread to the GI tract in invasive lobular carcinoma compared to invasive carcinoma of no special type is still unknown, although some authors assume it could be due to a particular tropism of lobular cells [10].

Schwarz et al. reported the median interval between the diagnosis of breast cancer and the development of GI tract metastasis to be 6 years [11]. Synchronous metastases can occur but are rare and can delay the diagnosis and make the detection of the primary breast cancer a challenge, as it was in our case.

Symptoms are usually dependent on the site of metastasis, with constipation, obstruction, tenesmus, and rectal bleeding which are more related to rectal metastasis. These symptoms can mimic primary GI tract pathologies like malignancies and inflammatory bowel disease, which are much more prevalent.

The best diagnostic methods are found to be colonoscopy and biopsy. Rectal metastatic lesions found on colonoscopy are usually diffuse leading to thickening and narrowing of the rectal lumen [12].

Histopathological diagnosis can be challenging in the absence of the diagnosis of primary breast cancer as it was in our case. In addition, the “signet-ring” morphology of lobular carcinoma may mimic other primary tumors, i.e., gastric carcinoma [13] and primary signet ring cell carcinoma with tubular adenoma of the rectum [14], and even rarer conditions such as synchronous primary colonic adenocarcinoma and metastatic lobular carcinoma as described in a case report [15] making the diagnosis more difficult.

On the other hand, metastases of lobular carcinoma have a certain fashion of spread with intramural infiltration growing within the serosal, muscular, and submucosal layers [16]. And last but not least, the lack of dysplasia of the rectal mucosa is often helpful in distinguishing between a primary and metastatic lesion [17].

Immunohistochemistry can be useful in establishing the diagnosis; cancerous breast cells are usually positive for CK-7, mammaglobin, GCDFP-15, estrogen, and progesterone receptors, although their positivity is not limited to breast carcinoma and was found positive in gastric carcinoma [18] and GATA-3 which has been recently used and noted to be positive in 100% of lobular breast cancers, compared to 5% of gastric cancers [19], while CK 20 and CEA are almost always present in primary colorectal tumors and absent in breast carcinomas. In this case, the rectal and peritoneal metastasis was positive for CK-7 and negative for CK 20 which matched the immunohistochemical findings of the primary breast cancer which, alongside the histological findings, favored the final diagnosis of metastatic lobular carcinoma.

Radiological findings usually mimic primary GI tract malignancies and are of less benefit in reaching the final diagnosis.

Due to the rarity of the condition, few data on treatment were found in the literature. Systemic treatment, i.e., hormonal and chemotherapy, is usually employed to patients with confirmed GI tract metastasis [20]. There were 2 cases reported by Tang et al. that showed settlement of intestinal obstruction due to metastatic lobular carcinoma with a parenteral endocrine hormonal agent (fulvestrant) [21]. Surgical intervention is used for palliative purposes in patients with intestinal obstruction, perforation, or bleeding or for diagnostic purposes.

Survival after diagnosis of GI metastases in breast cancer patients is poor with few patients surviving beyond two years [20].

## 4. Conclusion

GI tract metastasis from primary breast cancer is rare and not easy to diagnose. It should be suspected in a patient with a history of lobular breast carcinoma with new onset of GI tract symptoms, although synchronous tumors can rarely occur.

Histopathological and immunohistochemical findings are useful in detecting metastatic lesions.

Systemic treatment is used for patients with metastasis, with a poor survival rate.

Although it is considered a rare presentation, more unusual presentation of metastatic disease can be anticipated nowadays, due to the survival of breast cancer patients.
